# Potential Influences of the Darknet on Illicit Drug Diffusion

**DOI:** 10.1007/s40429-022-00439-2

**Published:** 2022-09-10

**Authors:** Angus Bancroft

**Affiliations:** University of Edinburgh, 18 Buccleuch Place, 4f1, Edinburgh, EH8 9JS UK

**Keywords:** Drug diffusion, Digital society, Darknet, Cryptomarkets, Drug dealing

## Abstract

**Purpose of Review:**

Darknet-hosted drug markets (‘cryptomarkets’) are an established model of illicit drug distribution which makes use of specialised online hosting and payment systems to link buyers and sellers remotely. Cryptomarkets appear to professionalise, gentrify and integrate drug markets. Therefore, they can be hypothesised to have effects on drug availability by allowing purchases by people who use drugs (PWUD) outside of face-to-face networks that have typified drug distribution. They may attract new buyers and may change use patterns by offering a greater range of higher-potency drugs. This paper examines the research on cryptomarkets’ potential impacts on drug availability.

**Recent Findings:**

1. Cryptomarkets tend to address established PWUD who mainly already have access to existing distribution systems. Their greatest impact may be on what is available and the quantities available, and not the overall ease of access.

2. Cryptomarkets may provide new data sources which can inform our understanding of drug markets.

3. Cryptomarkets may define PWUD as consumers and contribute to reshaping their identities around principles of self-directed, informed consumption.

4. In terms of size, cryptomarkets are currently smaller than other modes of digital drug distribution such as through social media and messaging apps and should be seen as a specialist subset of that genre.

5. Users of cryptomarkets often integrate drug-purchase and consumption repertoires across multiple sites, online and offline, and cryptomarkets can be one element.

**Summary:**

The cryptomarkets are of interest partly because they alter the practical calculus around drug diffusion and partly because they contribute to the formation of digitally enabled drug use which emphasises a consumer relationship between buyer and seller.

## Introduction

The availability of controlled substances is mediated through two broad and interrelated distribution types. Social supply between friends and acquaintances relies on a moral economy of sharing and reciprocity [1]. Transactional commercial supply in contrast emphasises profits and market-mediated relationships, and sometimes validates predation and exploitation [2]. Digital modes of drug distribution reshape both these distribution forms. The internet is a modern bazaar [3] of drug-selling modes, expanded psychoactive repertoires [4] and places of community harm reduction, which revise dominant narratives of drug use and PWUD [5]. One innovation has been the emergence of online cryptomarkets. These are specialised markets hosted anonymously using the Tor darknet [6]. Tor is an internet service which protects those who use it from monitoring and promotes anonymity through data routing and encryption. It also permits services to be hosted anonymously, using what are called onion or location-hidden services. A server can be connected to the Tor network without its location being detected.

Cryptomarkets are Tor-connected services which allow the exchange of illicit goods and services. Most of them present as shopfronts where vendors sell an array of drugs. Buyers pay using a cryptocurrency, typically Bitcoin, and the drug is delivered to them through the postal or courier system. Buyers are encouraged to leave reviews of the product and the vendor. Lively discussion forums discuss the quality of the drugs sold and the professionalism of vendors, among other topics.

**Fig. 1:**
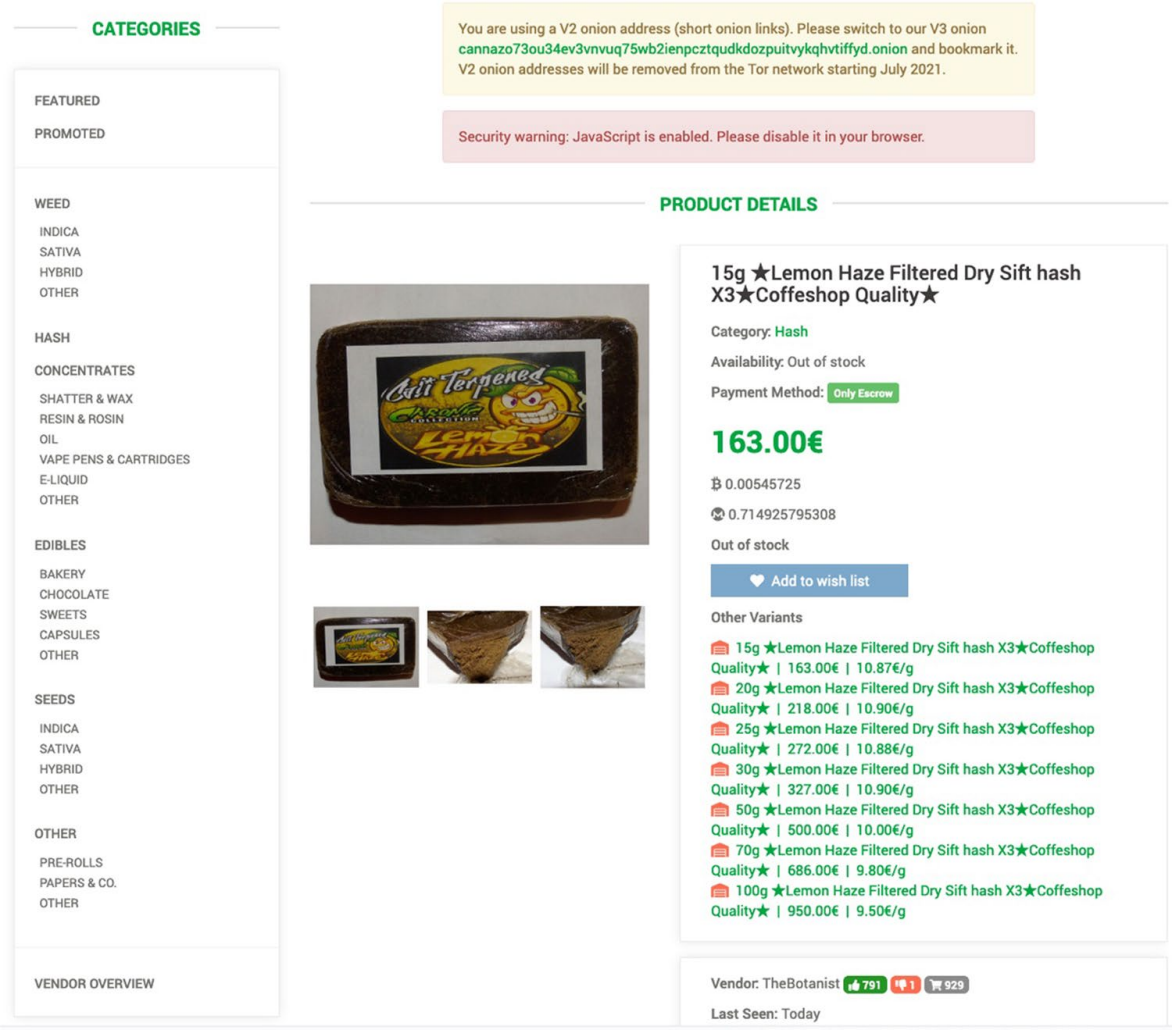
Vendor listing from a darknet cryptomarket*

Figure 1 shows a listing from a market specialising in cannabis sales. The listing typifies the way in which drugs are presented for sale. The vendor in this case ships from Spain and offers shipping within the European Union. Charges are added for express shipping. Discounts are provided for larger orders. The seller-buyer relationship is remote and impersonal, and the market is public and open, with features designed to promote professionalism. The design is typical of the kind of cryptomarket in common use in Western Europe, North America and Australia. It contrasts with Hydra, the Russian language market, which is more formalised and monopolistic, and which controls a high proportion of the Russian the drug market [7].

Cryptomarkets are also the focus of methodological innovation. Due to their open design, cryptomarkets have facilitated the emergence of new digital trace methods to track changes in drug markets such as the DATACRYPTO crawler maintained by David Décary-Hétu [8] and the application of large data analysis using machine learning [9]. These innovations allow for early confirmation of market changes such as the emergence of fentanyl and other novel synthetic opioids [10, 11] and of new drug delivery systems, such as e-cigarette/vaping methods for drug consumption [12].

## The Emergence and Each of Cryptomarkets

Cryptomarkets emerged in 2011 with the launch of Silk Road on the Tor network. Silk Road’s openness and anonymity signalled the arrival of a new type of drug diffusion [13]. It also signalled a new paradigm for drug markets that has since been emulated in other venues [14–16], one that emphasises information dense rationality in exchange [17].

After Silk Road was shut down by law enforcement, many other markets proliferated, sparking rounds of innovation and disruption between market administrators and law enforcement [18]. Disruption tended to demonstrate the resilience of the illicit drug market ecosystem [19]. Law enforcement takedowns of cryptomarkets stimulated a process of reconfiguration in the market [20•]. There is a tendency for informational matrices to degrade quickly. At one point, the cryptomarkets were served by a range of sites/forums such as the cross-site aggregator grams, the r/DarkNetMarkets Reddit forum and the DeepDotWeb site. However, this information ecosphere has been hampered as DeepDotWeb was shut by police, and r/DarkNetMarkets was closed by Reddit.

Recent estimates put the cryptomarkets as a substantial but definite minority of the drug market overall, worth around €750,000 Euro per day for sites serving European locations [21]. The Global Drug Survey records steady growth in use among its respondents, from 4.7 in 2014 to 15% in 2020 obtaining at least some of their drugs from darknet sites in the previous 12 months [22]. Products sold range widely, with an emphasis on cocaine, cannabis, novel psychoactive substances, sedatives and stimulants. Most illicit drugs are available in some form, but the product balance tends towards the ‘psychonaut’ profile, those who use drugs for self-exploration [23]. Alongside that, there are many self-identified dependent and addicted PWUD who find the predictability, professionalism and stability of supply a significant benefit [24].

The cryptomarkets are part of an ecosystem of messaging apps, webpages, discussion servers and social media platforms that service drug markets, mainly based in Europe, North America and Australasia [25•]. They serve the end point of the global trafficking network, supplementing and sometimes replacing the trafficker to supplier/buyer stage [26] in consumer countries [27] and excluding the global south [28]. Though sometimes depersonalised, they are evolving and provide the basis of dealer-to-buyer direct dealing [29•]. The cryptomarkets may be best seen as one part of a larger flexible social and technological structure that facilitates rapid arrangement of deals between parties and expands the range of drugs sold. Drug sellers and buyers navigate within cryptomarkets depending on the changing landscape and their specific requirements. This system generates an informal feedback loop allowing dealers to make more rapid decisions about what segments of the market to service.

## Effect on Purchases and Drug Diffusion

Cryptomarkets are designed to expose specific attributes of the drug being sold. Depending on the valued characteristics of the substance, these might be the intoxication effect, texture, smell, appearance, potency, ease of titration, activity in combination with other substances and pharmacokinetic behaviours. Generically, these are referred to as quality, which means many different things to different people [30]. Whether and in what way the specific drug being sold is effective is the subject of extensive discussion on each market’s associated forums. The informational context is supplemented by the use of independent drug-checking services by vendors and buyers. Though this can mislead and give people a false sense of security, it may normalise drug-checking as an expected part of drug-sale-and-consumption cycles [31].

Cryptomarkets also expose pricing, allowing buyers to compare offers from different suppliers. Pricing dynamics are similar to face to face markets, with bigger quantities meaning better deals. Pricing may reflect the ability of more successful vendors to command more lucrative prices due to claimed higher quality and greater security, leading to a price/quality ramp [32]. Higher prices may also reflect a premium for perceived safety of the buying process and quality of the product, demanding a comfort premium in addition to the normal risk premium incorporated into illicit drug sales [33]. On the other hand, research has found little difference between prices commanded on cryptomarkets compared to those on social media [34]. Therefore, we can see immediately that cryptomarkets promote particular kinds of market relationships between buyers and sellers: a focus on quality, competition, safety for both parties, greater choice and a tendency towards promoting high-value, bulk buys [14]. They promote what often matters to participants: reputation, displayed materials and socially remote interactions primarily focused around the market relationship. Emphasising the individualising nature of the market, one-to-one relationships are often more significant than community reputation [35].

One impact is to foreground each drug being sold as a specific branded consumer product with pharmacological attributes that can be closely assayed. This process thus draws on and brings together people’s cumulative experiential and subcultural knowledge, in common with other online drug-focused forums that discuss not just the quality of each drug, but what the drug is to them as a categorical object [36]. Behaviour is also changed. Easier availability may reduce temptations to hoard [37], but tendencies towards vendors selling solely or at discounted rates in larger quantities may counteract that. The benefits of making large purchases means that purchases are often made with the intent of social supply [38]. They also may alter the context of use. Some cryptomarket users exhibit more isolated use patterns such as using MDMA/Ecstasy, cocaine and LSD alone [39]. Ease of access also alters localised drug cultures, with for example 2-CB becoming more common in some rural areas of Scotland due to darknet access.

Most users of the cryptomarkets are not novices and already have established experience in face-to-face markets. Individuals may be attracted by predictable supplies, choices and perceptions of reduced risk. Users are predominantly male and young [40]. Some events such as COVID-19 pandemic-related lockdowns seem to have drawn large numbers of new PWUD into the darknet [41]. Many new entrants may just as quickly leave when they find the cryptomarkets do not suit their needs. Successful users of cryptomarkets often need to learn and socialise themselves into the system to make it work to good effect. The technical challenges and cultural barriers to entry may make them self-limiting to an extent [42].

Cryptomarkets are a focus for the gentrification hypothesis which proposes that a combination of long-established social, economic and technical conditions is serving to reduce the importance of violence and predation in drug distribution [17, 43]. Drug delivery has displaced street- or house-based exchanges in some circumstances; drug markets have become segmented by class and race; and the opportunities for combining drug dealing with other vice-exploitation crimes has declined [44]. Cryptomarkets extend some of these developments, seeking to emphasise conflict resolution, cooperation and professionalism and punish predation [45, 46], making their ethos more attractive to buyers and dealers [47]. That may serve to reduce some of the collateral harms of the illicit drug market [48] while at the same time concentrating risk and systemic violence among an already marginalised segment of the drug-using population that has little access to drug-delivery methods. While the cryptomarkets do put gentrification to the fore, they also shift power in the marketplace and create new opportunities for vendors to develop exploitative or coercive strategies and techniques [49].

As much as effective changes in the operation of the drug market, cryptomarkets have been part of a generation shift towards PWUD integrating drug-purchase and consumption repertoires across multiple platforms, online and offline, of which cryptomarkets can be one element. They also emphasise innovation taking place in other related technological domains. Televend is an example of an automated system that uses the Telegram app to mimic some attributes of cryptomarket systems [50]. Tor darknet forums become meeting places for dealing to occur on social media rather than in the cryptomarkets [51]. Internet platforms are used to create matrices of territories, delivery methods and relationships through which buyers and sellers may operate. The context is a general expansion of convenience, with changed spatial/territorial supply practices [52] and adaptive social/technical networks [53]. They do not override limits of territory and national borders, but transpire within them [54]. Cryptomarkets are currently smaller than other modes of digital drug distribution such as through social media and messaging apps and should be seen as a specialist subset of that distribution type, which adopts and shares the same gentrified, rational utilitarian stance.

## Conclusion: the Shifting Territory of the Digital Drug Market

Cryptomarkets are part of an evolving trend where communities of PWUD adapt and develop technological systems to their ends. The cryptomarket distribution system is emblematic of the move to drug distribution by delivery, whether through the postal system or tailored distribution services. They may now be being superseded in technical prowess by well-crafted, custom-built systems that use messaging apps [55, 56] and rather than representing the future are an established and stable if evolving niche. As a whole, these systems sometimes augment and sometimes bypass face-to-face markets and therefore may not be immediately open to the kind of incidental interventions that harm-reduction services may make. Adaption is needed and has been demonstrated in order to reach PWUD [57]. Having said that, user people will be consuming at places where services may be present, such as raves and festivals, but the rise of at-home delivery means that both distribution patterns and locations of consumption are changing.

Consumption may occur much more at home, especially with the impact of the COVID-19 pandemic globally [58]. The pandemic has affirmed and extended existing inequalities [59], and the digital market has contributed to that. Individuals who are more affluent and better connected have often continued drug consumption with little interruption. Those who do not have access to these distribution modes have often pursued shifting and sometimes predatory street markets. The impact of the darknet has to be fully seen in this context, as one component of an evolving social-technical infrastructure for drug distribution and consumption may include harm-reduction advice such as drug-checking services [60].
